# Actividad antimicrobiana de hongos endófitos de las plantas medicinales *Mammea americana* (Calophyllaceae) y *Moringa oleifera* (Moringaceae)

**DOI:** 10.7705/biomedica.4644

**Published:** 2020-03-30

**Authors:** Wilmer Giovanny Mosquera, Libeth Yajaira Criado, Beatriz Elena Guerra

**Affiliations:** 1 Grupo de Investigación en Biotecnología, Agroambiente y Salud - Microbiota; maestría en Investigación de Enfermedades Infecciosas, Universidad de Santander, Bucaramanga, Colombia Universidad de Santander Universidad de Santander Bucaramanga Colombia; 2 Grupo Salud Comunid-UDES, Escuela de Medicina, Universidad de Santander, Bucaramanga, Colombia Universidad de Santander Universidad de Santander Bucaramanga Colombia

**Keywords:** farmacorresistencia microbiana, endófitos, plantas medicinales, Escherichia coli, Staphylococcus aureus, Drug resistance, microbial, endophytes, plants, medicinal, Escherichia coli, Staphylococcus aureus

## Abstract

**Introducción.:**

Las enfermedades infecciosas son una causa importante de muertes en el mundo. La resistencia antimicrobiana es un problema global, por lo que es conveniente la investigación de nuevas fuentes de agentes antimicrobianos de origen natural potencialmente efectivos.

**Objetivo.:**

Evaluar la actividad antimicrobiana de hongos endófitos de *Mammea americana* y *Moringa oleifera* en la cepa sensible (ATCC 29213) y en la cepa resistente (USb003) de *Staphylococcus aureus,* así como en la cepa sensible (ATCC 25922) y la cepa resistente (USb007) de *Escherichia coli.*

**Materiales y métodos.:**

Se aislaron 14 hongos endófitos de las hojas, semillas y tallos de las dos plantas en estudio. Se evaluó su actividad antimicrobiana mediante la formación de halos de sensibilidad por ensayo dual *in vitro* y pruebas con extractos etanólicos crudos provenientes de los endófitos a los que se les evaluó la concentración mínima inhibitoria (CMI), la concentración bactericida mínima (CBM) y la citotoxicidad.

**Resultados.:**

Tres extractos etanólicos de *Penicillium* sp., *Cladosporium* sp. (001) *y Cladosporium* sp. (002) presentaron mayores halos de inhibición en cepas sensibles y resistentes de *E. coli* y *S. aureus.* La CMI y la CBM halladas fueron estadísticamente significativas (p≤0,05), comparadas con el control de gentamicina. Las pruebas de citotoxicidad (concentración citotóxica, CC_50_>1.000) demostraron que los hongos endófitos poseen características bactericidas y no ocasionan daño alguno.

**Conclusión.:**

Se halló una fuente de metabolitos secundarios activos con propiedades antimicrobianas y no tóxicas en los hongos endófitos de *M. oleifera y M. americana;* estos hallazgos son importantes para continuar con la identificación química de los compuestos y el estudio de sus mecanismos de acción en estas plantas en las que el aislamiento de endófitos ha sido escaso.

## Abstract (en)

**Introduction:**

Infectious diseases represent one of the leading causes of death worldwide. Considering the growing global challenge of antimicrobial resistance, research into new sources of potentially effective antimicrobial agents from natural origins is of great importance for world health.

**Objective:**

To evaluate the antimicrobial activity of endophytic fungi from *Mammea americana and Moringa oleifera* upon *Staphylococcus aureus* (ATCC 29213), *S. aureus* (resistant strain USb003), *Escherichia coli* (ATCC 25922), and *E. coli* (resistant strain USb007).

**Materials and methods:**

We isolated endophytic fungi from the leaves, seeds, and stems of the two plants under study. We evaluated their antimicrobial activity through the formation of sensitivity haloes in dual tests *in vitro,* as well as in trials using crude ethanolic extracts from the endophytes. The minimum inhibitory concentration (MIC), minimum bactericidal concentration (MBC), and cytotoxicity o the substances were analyzed.

**Results:**

Three ethanolic extracts of *Penicillium* sp., *Cladosporium* (001), and *Cladosporium* (002) exhibited the greatest inhibition halos in sensitive and resistant strains of *E. coli* and S. *aureus.* The MIC and CBM found were statistically significant (p≤0.05) compared with the gentamicin control. Furthermore, the cytotoxicity test results of CC50>1,000 demonstrated that the endophytic fungi studied exhibit bactericidal characteristics without causing unintended damage.

**Conclusion:**

The endophytic fungi *M. oleifera* and *M. americana* represent a source of active secondary metabolites with antimicrobial and non-toxic properties. In light of these findings, further research should proceed with chemical identification of the compounds and the study of their mechanisms of action, especially given the paucity of current scientific knowledge concerning the isolation of endophytes in these plants.

La resistencia a los antibióticos es uno de los temas más importantes en salud pública a nivel mundial y su incremento incide en la prevalencia de las enfermedades infecciosas. La resistencia a los antibióticos ocurre ya sea por mutaciones o por adquisición de genes que la confieren mediante transferencia horizontal, siendo esta última el factor más importante ^1^. Se sabe que, históricamente, las plantas han tenido un papel importante como fuente natural de compuestos con actividad antimicrobiana; sin embargo, se estima que solo se ha investigado entre el 10 y el 15 % de las especies de plantas superiores que la exhiben ^2^. Hoy se reconoce que un gran número de los compuestos bioactivos de las plantas son producidos por las comunidades microbianas endófitas albergadas en sus tejidos ^3^.

La presencia de hongos endófitos se reportó por primera vez en 1898, aunque el concepto de 'endófito' ya se había descrito en 1866 ^4^, sin que en ese momento se reconociera su importancia. Años más tarde comenzaron a ser objeto de investigación y se registró su capacidad para colonizar tejidos de plantas sin causar enfermedad ^5^.

Estas comunidades de microorganismos endófitos constituyen un importante campo de investigación farmacológica por la producción de metabolitos secundarios, agentes antimicrobianos que contribuyen a la disminución de las infecciones causadas por agentes patógenos adyacentes. En la relación entre los hongos endófitos y las plantas se ha verificado una producción activa de metabolitos secundarios segregados por los dos tipos de organismos: así, el vegetal proporciona defensas y el hongo fomenta la producción de metabolitos de características virulentas como los fitotóxicos, y algunas enzimas dañinas como las exoenzimas. Esta relación se conoce como endofítica y, si no es equilibrada, el hongo actúa como agente patógeno, dada su capacidad virulenta ^6^^,^^7^. Se ha subrayado que las interacciones entre los endófitos y las plantas que los alojan contribuyen a la producción conjunta de diversas moléculas bioactivas ^8^.

En un número considerable de publicaciones, se ha dado a conocer la importancia de estos microorganismos en la agricultura y en la medicina. Aunque muchos de ellos no se han estudiado a fondo, sí han evidenciado su condición de portadores de agentes productores de metabolitos bioactivos útiles para la defensa de sus huéspedes frente a los agentes patógenos y como reservorios de diversidad genética, habiéndose comprobado que sus metabolitos se activan al interactuar con las vías metabólicas de su huésped ^9^.

Se ha demostrado que los metabolitos secundarios biosintetizados por hongos endófitos poseen una gran actividad biológica, lo que evidencia su relevancia como agentes fitotóxicos y antimicrobianos, y amerita que se siga explorando e investigando sobre nuevos metabolitos activos producidos por los diferentes microorganismos endófitos de tejidos vegetales, en especial de plantas tropicales.

*Mammea americana* L se conoce en América como una planta medicinal. Se cultiva en las Bahamas y, en menor escala, en Venezuela, Guyana, Surinam, Guyana Francesa, Ecuador y el norte de Brasil. En Colombia, se encuentra en los bosques seco tropical, húmedo tropical, húmedo premontano y muy húmedo premontano ^10^, y se la conoce como mamey de Cartagena de Indias, o como *Mammeeapple*^11^. La madera es de beneficio económico y sus frutos, de buen sabor y gran valor nutricional, se consumen en varias regiones, y se componen de 18 % de cáscara, 20 % de semilla y 62 % de pulpa. *Mammea americana* se usa en la medicina alternativa para el tratamiento de las parasitosis, la flema, el exceso de ácido úrico, la fiebre, la hipertensión arterial, las infecciones de la piel, el raquitismo y la pérdida de cabello, entre otros. Posee compuestos cumarínicos (cumarinas de *Mammea),* a los que se les han atribuido propiedades antioxidantes, antimicrobianas, insecticidas, anticancerígenas, antitumorales y antifúngicas ^12^, a pesar de lo cual poco o nada se han estudiado y aislado sus hongos endófitos.

Otra planta medicinal de importantes propiedades es *Moringa oleifera* Lam, originaria de Kerala, India, que ha sido introducida en varias partes del mundo y en Suramérica desde México hasta Perú, Paraguay y Brasil ^13^. Se le considera un árbol capaz de adaptarse bien a diferentes climas, aunque es más frecuente en climas templados y tolera menos las bajas temperaturas. Es muy resistente a los diferentes agentes patógenos que la puedan afectar y sus hojas poseen una gran cantidad de proteínas, vitaminas, minerales y aminoácidos ^14^. El aceite de sus semillas se utiliza mucho por su contenido de ácido oleico (73 %), similar al de la oliva. En sus semillas se han encontrado coagulantes naturales, y se utilizan para la limpieza y aclaramiento de aguas, por lo que se emplea para mejorar las condiciones sanitarias en algunos países menos desarrollados ^15^.

*Moringa oleifera* ha sido objeto de estudios por la actividad antimicrobiana de sus endófitos, aislados de diferentes partes de la planta, los cuales se encuentran en comunidades fúngicas ^16^^,^^17^ y en comunidades bacterianas. En varios estudios se ha propuesto su utilización con fines nutricionales y medicinales, sin embargo, poco se sabe de ellos y de la obtención de metabolitos activos potencialmente útiles como antimicrobianos. Los metabolitos secundarios que más se han obtenido de *M. oleifera* han sido los flavonoides, los flavonoles, las antocianinas, los polifenoles, los alcaloides y los taninos ^18^, todos de gran importancia en la agricultura y en la medicina.

La Organización Mundial de la Salud (OMS) ha incluido a *Escherichia coli* y *Staphylococcus aureus* en el grupo más importante de bacterias causantes de infecciones y resistencia a fármacos. *Escherichia coli* coloniza el sistema gastrointestinal de seres humanos y otros animales, y está implicada en una serie de enfermedades que incluyen infecciones de las vías urinarias, septicemia, neumonía, meningitis neonatal, peritonitis y gastroenteritis ^19^. Es agente causal de un porcentaje importante de la enfermedad diarreica en el mundo y se estima que ocasiona, aproximadamente, 775.000 muertes al año, principalmente entre la población infantil de los países menos desarrollados ^20^. Por su parte, las infecciones de las vías urinarias afectan a pacientes sintomáticos y asintomáticos ^21^, con gran prevalencia en mujeres y niñas.

Las cepas patógenas de *E. coli* han demostrado su capacidad de resistencia a diversos fármacos de los empleados para combatirla ^22^. Se han descrito varios mecanismos de resistencia (entre ellos, desvío de una etapa metabólica, alteración de blancos, inactivación enzimática y disminución de la acumulación intracelular del antimicrobiano) a medicamentos como la tetraciclinas, los betalactámicos, el cloranfenicol, las sulfonamidas, el trimetoprim y los aminoglucósidos ^23^.

*Staphylococcus aureus* se encuentra principalmente en los tejidos de la piel sana y, por lo general, no provoca daño alguno; sin embargo, puede generar infecciones en pacientes inmunosuprimidos cuando se pierden las barreras naturales o en caso de trauma, siendo una de las principales causas de infección en sitios quirúrgicos y en infecciones asociadas con la atención en salud ^24^. Varias cepas de *S. aureus* han evolucionado gradualmente y sobreviven a la acción de los betalactámicos, como oxacilina, meticilina y nafcilina, y de otro tipo de antibióticos ^25^. En algunos estudios se ha demostrado que dicha resistencia está regulada y codificada por casetes genéticos cromosómicos.

En los países desarrollados, se considera que la concentración inhibitoria mínima (CMI) de la vancomicina es de ≥16 µg/ml; si esta es de 2 a 8 µg/ml, la resistencia es moderada, y hay sensibilidad si la CIM es de 2 µg/ml o menor ^26^, en tanto que el mecanismo de resistencia a los aminoglucósidos, las tetraciclinas y la eritromicina se da mediante plásmidos. La resistencia a la meticilina es una de las más importantes, pues para los pacientes infectados representa una probabilidad de mortalidad del 64 % ^27^.

El problema de la resistencia bacteriana ha incentivado la investigación de nuevas fuentes de antibióticos de origen natural. En dicho contexto, el objetivo de este trabajo se centró en el análisis de la actividad antibacteriana de los hongos endófitos aislados de dos plantas medicinales, *M. oleifera* y *M. americana,* siendo la primera vez que la actividad antimicrobiana de esta última se reporta.

## Materiales y métodos

Se llevaron a cabo la búsqueda, el aislamiento y la caracterización a nivel de morfoespecie de hongos endófitos en hojas, tallos y semillas de *M. americana y M. oleifera.* Se procesaron, en promedio, 60 muestras vegetales para aislarlos y determinar su actividad antimicrobiana en las cepas sensibles (ATCC 29213) de S. *aureus* y (ATCC 25922) de *E. coli,* así como en las cepas resistentes (USb003) de S. *aureus* y (USb007) de *E. coli.* Se hicieron pruebas de concentración inhibitoria mínima (CMI), concentración bactericida mínima (CBM) y citotoxicidad en las cepas sensibles.

### Recolección de muestras vegetales

La recolección de tejidos vegetales (hojas, semillas y tallos) de *M. americana* y *M. oleifera* se hizo totalmente al azar en 20 árboles visiblemente sanos. Las muestras de *M. americana* se obtuvieron en el municipio de Turbaco, departamento de Bolívar (10°19'30''00 N y 1°17'29'' O), en la Costa Atlántica colombiana y, las de *M. oleifera,* en plantaciones de la finca Manzanares, localizada en el municipio de Floridablanca, Santander (07°06'35,6"N y 73°05'58"E).

### Aislamiento de hongos endófitos

Las muestras de los diversos tejidos de las dos plantas se cortaron en segmentos de 1 cm, se lavaron y desinfectaron superficialmente utilizando el método descrito por Unterseher, *et al.*^28^, con algunas modificaciones en los porcentajes y tiempos de acción del alcohol y el hipoclorito.

Cuatro segmentos vegetales se transfirieron de forma aséptica al medio de cultivo micológico agar de papa y dextrosa (APD) con suplemento de cloranfenicol a 100 ppm; luego se colocaron de forma equidistante y se incubaron a 30 ^a^C durante 20 días. El crecimiento de los endófitos se registró diariamente.

Para el aislamiento de colonias puras, se procedió a hacer repiques en nuevos medios micológicos a partir de las puntas de las hifas de las colonias fúngicas visiblemente diferentes y a sembrar en medio APD por triplicado con suplemento de 16 µg/ml de cloranfenicol a 25 °C, durante 15 días hasta obtener cultivos puros.

### Prueba de preselección de endófitos

La preselección de los endófitos con posible actividad antimicrobiana se determinó utilizando cepas sensibles de *S. aureus* (ATCC 29213) y de *E. coli* (ATCC 25922) y cepas de origen clínico resistentes a antibióticos de la colección de bacteriología de la Universidad de Santander: S. *aureus* (USb003) y *E. coli* (USb007). Se tomaron porciones de cada uno de los hongos endófitos aislados previamente en cultivos puros de agar papa-dextrosa y con ayuda de la punta de pipetas estériles, las porciones se transfirieron a la superficie de distintas placas de Petri que contenían el medio de cultivo Müller-Hinton. Estos medios de cultivo fueron previamente inoculados y sembrados con cada una de las bacterias en estudio, utilizando el método de Kirby-Bauer. Los cultivos duales (bacteria-hongo) se dejaron a 37 °C durante 12 a 18 horas para favorecer el crecimiento bacteriano; y la bioactividad de los hongos se evalúo seleccionando aquellas cepas que produjeron mayor halo de actividad.

Todas las pruebas se hicieron por triplicado para comparar el valor de las medias de los halos de cada uno de los endófitos aislados.

### Identificación de los endófitos

Los hongos endófitos que mostraron actividad en la prueba de preselección con base en los halos, se identificaron hasta el nivel de morfoespecie según sus características macroscópicas (color y aspecto de las colonias tanto en el reverso como el anverso, presencia de pigmentos que se difunden en el medio) y características microscópicas, tales como estructuras de reproducción asexual, aparición de conidias, y tamaño, forma y color de las esporas, observadas con un microscopio Nikon (Eclipse-Ni-u)™ a 10X y 40X.

Se utilizaron claves internacionales ^29^^,^^30^ disponibles en línea para la identificación de los endófitos hasta el nivel de morfoespecie.

### Obtención de extractos etanólicos fúngicos y pruebas de actividad antimicrobiana

Siguiendo los protocolos estandarizados para la obtención de biomasa de hongos endófitos utilizados en el Laboratorio de Micología de la Universidad de Santander, se consideró un promedio de 30 cultivos en APD y un crecimiento del micelio de 15 a 20 días, para obtener biomasa de cada una de las cepas fúngicas preseleccionadas.

Para la preparación inicial de soluciones madre de extracto etanólico crudo de endófitos, se consideró una relación entre peso y volumen (P/V) de 1:1 de biomasa y etanol; posteriormente, se agitó durante las primeras 24 horas, al cabo de las cuales se detuvo la agitación y los extractos se llevaron a tres partes de etanol y una de biomasa fúngica (3:1) para, luego, continuar la agitación durante 48 horas más. A continuación, se mantuvieron refrigerados en frascos de vidrio ámbar durante 15 días hasta su separación con el método de filtración a través de una membrana Millipore de 22 µm. Posteriormente, el filtrado se llevó al evaporador rotativo (Rotavapor R-300™ - Buchi). Los extractos secos se pesaron y almacenaron a 4 °C hasta su posterior utilización.

### Determinación de la actividad antibacteriana de los extractos de hongos crudos

La actividad antibacteriana de los metabolitos secundarios extraídos de los hongos endófitos de *M. americana y M. oleífera,* se analizó frente a las bacterias sensibles y resistentes a antibióticos mediante el método de difusión del pozo de agar. Los cultivos bacterianos se extendieron en placas de agar Müller-Hinton, luego se perforaron los pozos en las placas de agar y se vertieron en microplacas tres concentraciones del extracto crudo de 35 µl, 70 µl y 105 µl. La actividad antibacteriana se detectó después de una incubación de 24 a 48 horas a 37 °C. La presencia de una zona de aclaramiento en las placas, se usó como indicador de la naturaleza antibiótica y bioactiva de los extractos de endófitos fúngicos. Como control positivo se usó gentamicina y, como control negativo, dimetilsulfóxido (DMSO). Se llevaron a cabo tres réplicas de cada prueba de actividad antibacteriana.

### Determinación de las concentraciones inhibitoria y bactericida mínimas

Las concentraciones mínimas, inhibitoria (CIM) y bactericida (CBM), de los extractos etanólicos crudos, se determinaron mediante microdilución según las normas del *National Committee for Clinical Laboratory Standards* (NCCLS, 2004), y se evaluaron en las cepas *E. coli* (ATCC 25922) y S. *aureus* (ATCC 29213), siguiendo lo sugerido por el *Clinical and Laboratory Standards Institute* (CLSI).

Después de la incubación bajo condiciones apropiadas, la concentración más baja de extracto que inhibió el crecimiento visible de cada una de las bacterias en estudio, se registró como la CIM, en tanto que la concentración bactericida mínima (CBM) se halló a partir de las pruebas de CIM mediante dilución en caldo de cultivo secundario en placas de agar. La gentamicina se usó como agente antibacteriano estándar.

Los experimentos se hicieron por duplicado en cada placa y en tres momentos diferentes con los extractos etanólicos de los hongos que presentaron mayores halos de inhibición en las bacterias evaluadas.

### Pruebas de citotoxicidad

Los extractos fúngicos con mayor actividad inhibitoria en cada una de las bacterias evaluadas *in vitro,* se sometieron a las pruebas de citotoxicidad utilizando la línea celular VERO (células de riñón de mono verde: *Cercopithecus aethiops,* ATCC: CCL-81). Los cultivos se mantuvieron con medio esencial mínimo de Eagle modificado por Dulbecco (DMEM) y suero fetal bovino al 5 %, y se incubaron a 37 °C en 5 % de CO_2_ en cajas de cultivo celular Nunc™ de 25 ml. Se trasladaron a una placa de 96 pozos de 4,0 x 10^5^ células por pozo y se agregaron 100 µl de los extractos fúngicos en concentraciones de 4.000 µg/ml en DMSO al 10 % v/v y en dilución seriada hasta los 400 µg/ml, y se mantuvieron en iguales condiciones de suplementos e incubación durante 72 horas, al cabo de las cuales se contaron las células viables por exclusión con azul de tripano. En cada caso se determinó la concentración citotóxica media (CC_50_), la cual indica la reducción del número de células viables al 50 %.

Como control negativo, se utilizó el cultivo celular sin adición de la muestra, al cual también se le hizo conteo celular como punto de comparación del crecimiento logarítmico a las 72 horas.

Todas las pruebas se llevaron a cabo por duplicado. En el análisis de los datos de citotoxicidad, se utilizó el programa estadístico MsxIfitTM (ID Business Solution, Guildford, UK) para calcular, mediante análisis de regresión sigmoidea, la concentración citotóxica media (CC_50_) de cada uno de los extractos etanólicos de hongos endófitos de *M. americana* y *M. oleifera.*

Los experimentos se realizaron por triplicado en tres momentos independientes y con tres extractos de hongos endófitos seleccionados, y la toxicidad se evaluó en las cepas ATTCC en estudio.

### Datos estadísticos

La actividad antimicrobiana de los extractos se evaluó con el programa estadístico Stata 14™, para determinar promedios, medianas, rangos y comparación de halos de sensibilidad en las bacterias (sensibles y resistentes) utilizadas en las pruebas. La prueba estadística t de Student se empleó para comparar los halos de inhibición en la preselección mediante ensayo dual, y las de Mann-Whitney y de Kruskal-Wallis, para comparar los halos de inhibición con cada uno de los extractos fúngicos, utilizando como control la gentamicina y considerando como referencia los datos de halos de sensibilidad y resistencia descritos en la literatura especializada.

## Resultados

### Porcentajes de aislamientos de endófitos

**Figura 1:**
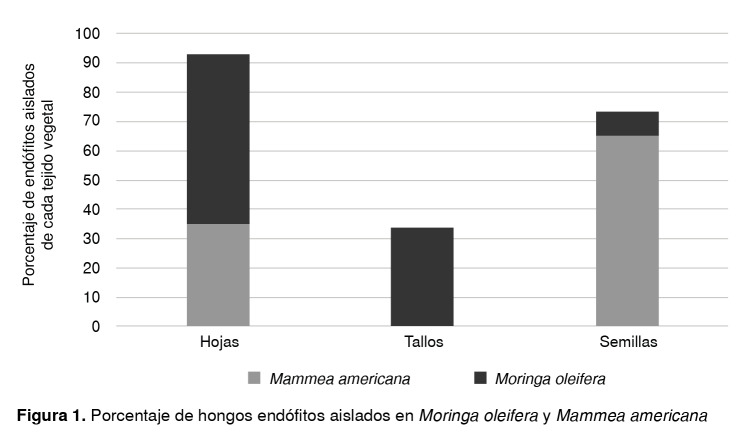
Porcentaje de hongos endófitos aislados en *Moringa oleifera* y *Mammea americana*

En *M. oleífera* se aislaron endófitos de los tres tejidos vegetales, con un porcentaje mayor en hojas (58 %); en los tallos fue de 34 % y en las semillas, de 8 %. En *M. americana,* por el contrario, la mayoría (65 %) de los endófitos se obtuvieron de la semilla, y el resto se obtuvo de las hojas (figura 1).

### Pruebas de preselección por ensayo dual

Las pruebas de preselección por ensayo dual *in vitro* con base en la formación de halos de inhibición del crecimiento bacteriano por parte de los hongos endófitos, se analizaron mediante la prueba t de Student, la cual permitió preseleccionar los 14 hongos endófitos (cuadro 1) aislados de los diferentes tejidos vegetales con los mayores halos de inhibición del crecimiento bacteriano en cepas sensibles y resistentes a antibióticos de *E. coli y S. aureus.*

**Cuadro 1:** Preselección por ensayo dual de la actividad antimicrobiana de hongos endófitos de *Mammea americana* y *Moringa oleifera* en cepas de *Escherichia coli* y *Staphyloccocus aureus* sensibles y resistentes

Hongos endófitos	Tejido de aislamiento de los endófitos	*E. coli* sensible (ATCC 29213)Tamaño de halos (mm)^**^	*E. coli* resistente (USb007) Tamaño de halos (mm)^**^	*S. aureus* sensible (ATCC 29213) Tamaño de halos (mm)^**^	*S. aureus* resistente (USb003) Tamaño de halos (mm)^**^
HESMa1	Semilla	25	13,6	25	24,3
HEHMo2	Hojas	28,3	13,3	28,3	25,3
HEHMo3	Hojas	24,3	17,6	25	23,6
HEHMa4	Hojas	23,3	16,3	23,6	23,6
HESMa5	Semilla	30,6	22,3	24,6	25
HEHMa6	Hojas	30,3	19	23,3	23,3
HETMo7	Tallo	25	11,6	29	28,6
HESMo8	Semilla	27,6	18,6	28,6	28
HETMo9	Tallo	27	17,3	26,3	26,6
HESMa10	Semilla	20,6	16,3	27,6	27,6
HEHMo11	Hojas	25,6	22,6	32,6	32
HEHMo12	Hojas	31,3	19,6	29	25,6
HESMa13	Semilla	30	21,6	25,6	25,6
HEHMa14	Hojas	14	12,6	26	26
Valor promedio		22	19	26,8	27,75

HE: hongo endófito; H: hoja; T: tallo; S: semilla; Mo: *M. oleifera;* Ma: *M. americana;* mm**: promedio del valor de halos en tres réplicas. Control de gentamicina en cepas sensibles de *S. aureus:* 22 mm. Control de gentamicina en cepas sensibles de *E. coli:* 23 mm. Control de gentamicina en cepas resistentes de *E. coli* y *S. aureus:* 00 mm

### Actividad antibacteriana de extractos crudos de endófitos según halos de inhibición

En los cuadros 2 y 3, se comparan los halos de inhibición del crecimiento bacteriano de las cepas sensibles y resistentes de S. *aureus* y *E. coli* con diferentes concentraciones de extractos etanólicos de hongos endófitos y el control de gentamicina (prueba estadística de Mann-Whitney).

**Cuadro 2:** Inhibición del crecimiento de las cepas sensibles y resistentes de *Staphylococcus aureus* con extractos de hongos endófitos, comparada con el control de gentamicina

Codificación fúngica	Extracto de hongos endófitos (µl)	***S. aureus* (ATCC 29213) mm mediana (min.-máx.)**	***S. aureus* (USb003) mm mediana (min.-máx.)**	Valor de p Sensible Vs. resistente	Valor de p Sensible Vs. control (gentamicina)	Valor de p Resistente Vs. control (gentamicina)**
HESMa1	35	0	0	-	0,0253	0,0253
HESMa1	70	0	0	-	0,0253	0,0253
HESMa1	105	0	0	-	0,0253	0,0253
HEHMo2	35	0	0	-	0,0253	0,0253
HEHMo2	70	0	0	-	0,0253	0,0253
HEHMo2	105	0	0	-	0,0253	0,0253
HEHMo3	35	13 (13 - 13)	0	0,0253	0,0253	0,0253
HEHMo3	70	22 (22 - 22)	0	0,0253	-	0,0253
HEHMo3	105	22 (22 - 22)	12 (12 - 12)	0,0253	0,0143	0,0253
HEHMa4	35	0	0	-	0,0253	0,0253
HEHMa4	70	0	0	-	0,0253	0,0253
HEHMa4	105	0	0	-	0,0253	0,0253
HESMa5	35	0	0	-	0,0253	0,0253
HESMa5	70	15 (15 - 16)	15 (15 - 15)	0,3173	0,0339	0,0253
HESMa5	105	17 (17 - 23)	20 (20 - 21)	0,6531	0,1213	0,0339
HEHMa6	35	12 (12 - 12)	11 (11 - 11)	0,0253	0,0253	0,0253
HEHMa6	70	18 (18 - 19)	18 (18 - 18)	0,1138	0,0339	0,0253
HEHMa6	105	22 (22 - 22)	21 (21 - 21)	0,0253	-	0,0253
HETMo7	35	0	0	-	0,0253	0,0253
HETMo7	70	0	0	-	0,0253	0,0253
HETMo7	105	0	0	-	0,0253	0,0253
HESMo8	35	15 (15 - 16)	12 (12 - 13)	0,0431	0,0339	0,0339
HESMo8	70	18 (18 - 19)	17 (17 - 18)	0,0431	0,0339	0,0339
HESMo8	105	20 (20 - 21)	21 (19 - 21)	0,3458	0,0339	0,0369
HETMo9	35	20 (20 - 20)	15 (16 - 15)	0,0339	0,0253	0,0339
HETMo9	70	23 (23 - 24)	24 (23 - 24)	0,0431	0,0339	0,1138
HETMo9	105	24 (24 - 24)	25 (18 - 25)	1	0,0253	0,4867
HESMa10	35	19 (19 - 19)	15 (15 - 16)	0,0339	0,0253	0,0339
HESMa10	70	20 (20 - 20)	21 (18 - 21)	1	0,0253	0,0369
HESMa10	105	20 (20 - 20)	20 (19 - 20)	0,3173	0,0253	0,0339
HEHMo11	35	0	0	-	0,0253	0,0253
HEHMo11	70	16 (16 - 16)	15 (15 - 15)	0,0253	0,0253	0,0253
HEHMo11	105	18 (18 -18)	15 (15 - 15)	0,0253	0,0253	0,0253
HEHMo12	35	0	0	-	0,0253	0,0253
HEHMo12	70	0	0	-	0,0253	0,0253
HEHMo12	105	0	0	-	0,0253	0,0253
HESMa13	35	0	0	-	0,0253	0,0253
HESMa13	70	0	0	-	0,0253	0,0253
HESMa13	105	0	0	-	0,0253	0,0253
HEHMa14	35	0	0	-	0,0253	0,0253
HEHMa14	70	0	0	-	0,0253	0,0253
HEHMa14	105	0	0	-	0,0253	0,0253

He: hongo endófito; S: semilla; T: tallo; H: hoja; Mo: *M. oleífera;* Ma: *M. americana* * Control de gentamicina en cepas sensibles: 22 mm; ** Control de gentamicina en cepas resistentes: 0,0. Los valores se hallaron por triplicado (prueba estadística de Mann-Whitney)

**Cuadro 3:** Inhibición del crecimiento de cepas sensibles y resistentes de *Escherichia coli* con extractos de hongos endófitos, comparada con el control de gentamicina

Código de hongo endófito	Extracto etanólico (μl)	***E. coli* sensible (ATCC 29213) (mm) Mediana (min.-máx.)**	***E. coli* resistente (USb007) (mm) Mediana (min.-máx.)**	Valor de p Sensible Vs. resistente	Valor de p Sensible Vs. control (gentamicina)*	Valor de p Resistente Vs. control (gentamicina)**
HESMa1	35	0	0	-	0,0495	-
HESMa1	70	16 (16 - 16)	16 (16 - 16)	-	0,0495	0,0253
HESMa1	105	18 (17 - 18)	17 (17 - 17)	0,11	0,0495	0,0253
HEHMo2	35	0	0	-	1	-
HEHMo2	70	0	0	-	1	-
HEHMo2	105	0	0	-	1	-
HEHMo3	35	0	0	-	1	-
HEHMo3	70	0	0	-	1	-
HEHMo3	105	0	0	-	1	-
HEHMa4	35	12 (11 - 12)	0	0,0339	0,0339	-
HEHMa4	70	16 (15 - 16)	17 (16 - 17)	0,099	0,0339	0,0339
HEHMa4	105	20 (15 - 20)	18 (18 - 18)	0,48	0,0339	0,0253
HESMa5	35	0	0	-	0,0253	-
HESMa5	70	0	0	-	0,0253	-
HESMa5	105	19 (19 - 19)	19 (18 - 19)	0,32	0,0253	0,0339
HEHMa6	35	17 (17 - 18)	17 (17 - 17)	0,32	0,0339	0,0253
HEHMa6	70	23 (22 - 23)	20 (20 - 22)	0,07	0,32	0,0339
HEHMa6	105	24 (24 - 25)	24 (23 - 24)	0,20	0,0339	0,0339
HETMo7	35	0	0		1	-
HETMo7	70	0	0		1	-
HETMo7	105	0	0		1	-
HESMo8	35	12 (12 - 12)	12 (11 - 12)	0,32	0,0253	0,0339
HESMo8	70	20 (19 - 20)	21 (17 - 21)	0,32	0,0339	0,0339
HESMo8	105	22 (22 - 22)	22 (20 - 22)	0,32	0,0253	0,0339
HETMo9	35	14 (13 - 14)	14 (12 - 14)	0,80	0,0339	0,0339
HETMo9	70	21 (21 - 21)	21 (20 - 21)	0,32	0,0253	0,0339
HETMo9	105	23 (23 - 23)	22 (22 - 22)	0,0253	--	0,0253
HESMa10	35	17 (17 - 17)	12 (12 - 13)	0,0339	0,0253	0,0339
HESMa10	70	20 (20 - 20)	20 (17 - 21)	1	0,0253	0,0369
HESMa10	105	20 (20 - 20)	20 (19 - 20)	0,32	0,0253	0,0339
HEHMo11	35	20 (20 - 20)	18 (18 - 18)	0,0253	0,0253	0,0253
HEHMo11	70	21 (21 - 21)	20 (20 - 20)	0,0253	0,0253	0,0253
HEHMo11	105	22 (22 - 22)	22 (22 - 22)	1	0,0253	0,0253
HEHMo12	35	12 (12 - 12)	0	0,0253	0,0253	-
HEHMo12	70	18 (18 - 18)	12 (11 - 12)	0,0253	0,0253	0,0495
HEHMo12	105	18 (18 - 18)	15 (14 - 15)	0,0253	0,0253	0,0495
HESMa13	35	0	0		0,0253	-
HESMa13	70	14(14 - 14)	14(13 - 14)	0,32	0,0253	0,0339
HESMa13	105	21(21 - 21)	20(20 - 21)	0,11	0,0253	0,0339
HEHMa14	35	0	0		0,0253	-
HEHMa14	70	12(12 - 15)	0	0,0253	0,339	-
HEHMa14	105	16(15 - 16)	0	0,0253	0,0339	-

He: hongo endófito; S: semilla; T: tallo; H: hoja; Mo: *M. oleífera;* Ma: *M. americana* * Control de gentamicina en cepas sensibles: 23 mm **Control de gentamicina en cepas resistentes: 0. Los valores se hallaron por triplicado (prueba estadística de Mann-Whitney)

Siete de los extractos de endófitos fúngicos estudiados como antibacterianos presentaron halos de inhibición para las cepas sensibles y resistentes de S. *aureus* (cuadro 2), en tanto que 11 cepas de *E. coli* (cuadro 3) mostraron actividad antimicrobiana, según se evidenció con la formación de halos de inhibición del crecimiento.

La actividad antimicrobiana de los extractos etanólicos fúngicos de los endófitos fue diferente para las cepas de *S. aureus* sensibles y para las resistentes, con un rango de halos de inhibición de 13 a 24 mm y de 12 a 25 mm, respectivamente, siendo estos valores estadísticamente significativos con relación al control de gentamicina (cuadro 2).

Un comportamiento similar se observó en las pruebas de inhibición del crecimiento bacteriano en las cepas de *E. coli* con los diferentes extractos fúngicos de los endófitos, los cuales presentaron halos de inhibición en rangos similares a los observados para las cepas de *S. aureus* (cuadro 3).

En cuanto a la actividad antimicrobiana de los extractos fúngicos de los endófitos en las cepas de S. *aureus,* los extractos HEHMa6, HESMo8, HETMo9 y HESMa10 evidenciaron actividad inhibitoria frente a las cepas sensibles y a las resistentes. En cuanto a *E. coli,* el endófito HEHMa6 presentó un halo de inhibición significativamente mayor (p=0,0339) en las dos cepas: la sensible y la resistente, en comparación con el control de gentamicina.

Por otro lado, al comparar los halos de inhibición de los tres tipos de concentraciones de cada hongo mediante la prueba estadística de Kruskal-Wallis, se observó que, frente a la cepa sensible de *E. coli* (ATCC 29213), los extractos etánolicos fúngicos de los endófitos tendieron a incrementar la inhibición al aumentar la concentración del extracto en la prueba (p=0,0563 y p=0,0672, respectivamente). Un comportamiento similar se observó al aumentar la concentración de los extractos fúngicos en las pruebas de inhibición del crecimiento de las cepas de *S. aureus.*

### Actividad antimicrobiana y citotoxicidad de los extractos etanólicos crudos derivados de hongos endófitos

Teniendo en cuenta los resultados arrojados por el análisis estadístico, se consideró continuar con las pruebas de concentración inhibitoria mínima (CIM), concentración bactericida mínima (CBM) y citotoxicidad de los extractos fúngicos de los endófitos identificados como HEHMa6, HESMo8 y HESMa10, correspondientes, respectivamente, a las morfoespecies *Penicillium* spp., *Cladosporium* sp., aislamiento 001, y *Cladosporium* sp., aislamiento 002. Todos los valores hallados en las repeticiones fueron coherentes, ya que arrojaron el mismo valor de CIM. La CIM y la CBM registraron los mismos valores absolutos, con lo que se pudo evidenciar que los extractos tuvieron una capacidad bactericida importante (cuadro 4).

**Cuadro 4:** Actividades antimicrobianas y citotoxicidad de extractos etanólicos crudos derivados de hongos endófitos (CIM, CMB y CC_50_; µg/ml)

Endófitos	*S.aureus* CIM	*E. coli* CIM	*S. aureus* CMB	*E. coli* CMB	Células Vero (CC_50_±SD)
Extracto	2.000 µl/ml	1.000 µl/ml	2.000 µl/ml	1.000 µl/ml	> 1.000
HEHMa6					
*Penicillium* ssp.					
Extracto	1.000 µl/ml	2.000 µl/ml	1.000 µl/ml	2.000 µl/ml	>1.000
HESMo8					
*Cladosporium* (001)					
Extracto	1.000 µl/ml	2.000 µl/ml	>4.000 µl/ml	1.000 µl/ml	>1.000
HESMa10					
*Cladosporium* (002)					
Gentamicina	1000 µl/ml	0,250 l/ml	0,125 µl/ml	0,250 µl/ml	

Los resultados se expresan en µg/ml y son el promedio de tres experimentos independientes. *S. aureus* (ATCC 29213), *E. coli* (ATCC 25922)

El control con gentamicina se consideró óptimo según los estándares del CLSI. La CIM requerida por las cepas microbianas con cada uno de los tres extractos etanólicos crudos registró diferencias significativas (p≤0,05), lo que indica que el extracto HEHMa6 *(Penicillium* spp.) tuvo mejor bioactividad frente a *E. coli.* Por el contrario, el extracto HESMo8 *(Cladosporium* spp.) tuvo mejor bioactividad en *S. aureus,* lo que podría obedecer a factores tales como la composición química de los extractos y su actividad selectiva para microorganismos Gram positivos y Gram negativos. La CBM coincidió con la prueba de CIM, para la cual se consideró una CBM de tres o menos unidades formadoras de colonia (UFC) por caja.

La citotoxicidad de los extractos evaluados en células de mamífero presentó concentraciones CC_50_ superiores a 1.000 µg/ml (cuadro 4), lo que indicó que los extractos HEHMa6, HESMo8 y HESMa10, obtenidos de los hongos *Penicillium* spp., *Cladosporium* sp. 001 y *Cladosporium* sp.002, respectivamente, no produjeron ningún tipo de daño celular, lo que confirma su utilidad para identificar posibles moléculas bioactivas de interés para su uso en el ser humano.

### Identificación de hongos endófitos

**Figura 2:**
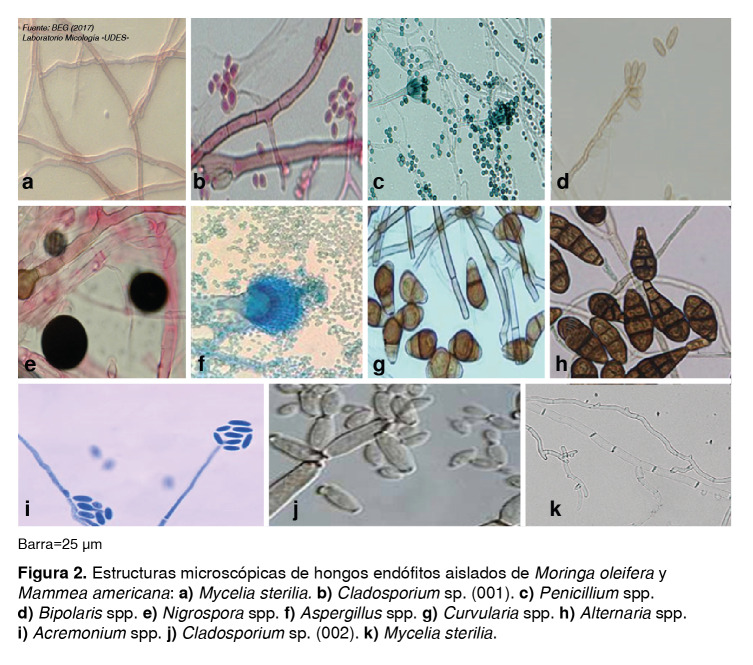
Estructuras microscópicas de hongos endófitos aislados de *Moringa oleifera* y *Mammea americana:* **a)** *Mycelia sterilia.* **b)** *Cladosporium* sp. (001). **c)** *Penicillium* spp. **d)** *Bipolaris* spp. **e)** *Nigrospora* spp. **f)** *Aspergillus* spp. **g)** *Curvularia* spp. **h)** *Alternaría* spp. **i)** *Acremonium* spp. **j)** *Cladosporium* sp. (002). **k)** *Mycelia sterilia.*

Los 14 hongos filamentosos aislados de los tejidos de *M. americana* y *M. oleifera* que presentaron actividad anti microbiana contra *E. coli* y S. *aureus,* se identificaron por sus características macroscópicas y microscópicas como miembros de las divisiones Ascomycota y Deuteromycota. Un alto porcentaje (85 %) de ellos se clasificó como miembro de Ascomycota y, de estos, un 75 % presentó pigmentos intrínsecos en sus hifas y esporas, por lo cual se clasificaron como Dematiáceos. El 25 % correspondió a hongos de hifas hialinas (cuadro 5). Los hongos clasificados como Deuteromycota se catalogaron como de micelio estéril, ya que sus géneros pertenecen a hongos imperfectos que no presentan esporulación y representaron solo el 15 % de los 14 hongos aislados con actividad antimicrobiana contra las dos bacterias ensayadas (cuadro 5 y figura 2).

**Cuadro 5:** Morfología de los hongos endófitos aislados de *Mammea americana* y *Moringa oleifera*

Código de aislamiento, clasificación por presencia o ausencia de pigmento en estructuras fúngicas	Género fúngico	Características macroscópicas y microscópicas
HESMa1 (Dematiáceo)	*Bipolaris* spp.	Colonias: color gris a café negruzco Reverso: negro Conidióforos: raquis ramificados, cortos, geniculados o en zigzag Conidios: elipsoidales, redondeados en ambos extremos y lisos
HEHMo2 (Dematiáceo)	*Mycelia sterilia*	Morfología similar a la de muchos hongos que se caracterizan por no producir ningún estado de conidios, sexual o asexual, reconocible en el cultivo. A nivel microscópico se observan hifas tabicadas con pigmentos oscuros; por conveniencia, se clasificaron como Mycelia sterilia.
HEHMo3 (Dematiáceo)	*Curvularia* spp.	Colonias: de crecimiento rápido, parecidas a la gamuza, de color marrón a marrón negruzco con reverso negro Conidióforos de forma recta, tabicados, conidios en sucesión simpodia Conidios de forma semilunar, redondeados en los extremos, de color marrón pálido, de 3 tabiques
HEHMa4 (Hialino)	*Aspergillus* spp. 001	Colonias: color verde claro con presencia de cleistotecio marrón rojizo oscuro Reverso: oliva a marrón púrpura Conidióforos: cortos, parduscos y lisos Conidios: globosos y de paredes rugosas
HESMa5 (Dematiáceo)	*Mycelia sterilia*	A nivel microscópico, se observan hifas con pigmentos oscuros, tabicadas y sin esporulación. Por conveniencia, se clasificaron como Mycelia sterilia.
HEHMa6 (Hialino)	*Penicillium* spp.	Colonias: verdes con periferia blanca Reverso: pardo a marrón Conidióforos: hialinos, lisos o de paredes rugosas Conidios: forman largas cadenas globosas en sucesión basípeta de una célula conidiógena especializada llamada fiálide.
HETMo7 (Dematiáceo)	*Alternaria* spp.	Colonias: de color negro a oliváceas. Reverso: pardo oscuro Conidióforos: ramificados, cortos Conidios multicelulares, oblicuos, café claro de paredes lisas
HESMo8 (Dematiáceo)	*Cladosporium* sp. 001	Colonias: verde oliva a negro Reverso: oliváceo a negro Conidios y conidióforos pigmentados, formándose en cadenas simples y ramificadas
HETMo9 (Hialino)	*Aspergillus* sp. 002	Colonias granulares, amarillo-marrones Reverso: marrón Conidióforos largos, vesícula globosa Fiálides que surgen en circunferencia, biseriadas Conidios redondos y pequeños que forman largas cadenas
HESMa10 (Dematiáceo)	*Cladosporium* sp. 002	Colonias: grises que se tornan negras, de aspecto similar a la gamuza Reverso: oliváceo-negro Conidios en cadenas acropetales ramificadas, equinuladas, de una a cuatro células
HEHMo11 (Dematiáceo)	*Nigrospora* sp. 001	Colonias: blancas al principio y, al cabo del tercer día, pardas a negras Reverso: negro Conidióforos: ramificados, incoloros a pardos Conidios: negros brillantes, solitarios, simples y esféricos
HEHMo12 (Hialino)	*Acremonium* spp.	Colonias: de color blanco a gris Hifas: delgadas, hialinas Conidios: unicelulares (ameroconidios), hialinos, alargados y algunos cilíndricos que forman agregados en cabezas viscosas en el ápice de cada filamento o conidióforo
HESMa13 (Dematiáceo)	*Nigrospora* sp. 002	Colonias: blancas al principio y, al cabo del tercer día, de color pardo a negro Reverso: negro Conidióforos: ramificados, incoloros a pardos Conidios: negros, solitarios, simples y esféricos
HEHMa14 (Dematiáceo)	*Mycelia sterilia*	Morfología similar a muchos hongos que se caracterizan porque no producen ningún estado de conidios, sexual o asexual, reconocible en el cultivo A nivel microscópico, se observan hifas con pigmentos oscuros, tabicadas. Por conveniencia, se clasificaron como *Mycelia sterilia.*

## Discusión

Se aislaron 14 hongos endófitos de *M. americana* y *M. oleifera* con capacidad de inhibir el crecimiento de *E. coli* y S. *aureus,* lo que evidenció su capacidad de sintetizar una amplia variedad de compuestos naturales bioactivos. En otros estudios de diferentes especies de plantas, se han aislado hongos endófitos con capacidad para inhibir agentes patógenos de animales y plantas, así como de producir diversos antimicrobianos ^31^^-^^35^. El 35 % de los endófitos reportados con actividad antimicrobiana se ha aislado de plantas medicinales ^36^, como las especies utilizadas en este estudio.

El ensayo dual (hongo-bacterias) realizado preliminarmente permitió una preselección de los hongos endófitos a partir de su capacidad de formar halos de inhibición del crecimiento de S. *aureus* y *E. coli,* tanto en cepas ATCC sensibles como en cepas clínicas resistentes a antimicrobianos. Todos los hongos preseleccionados evidenciaron actividad antimicrobiana; el promedio del tamaño de los halos de inhibición fue igual o mayor de 22 mm, excepto contra la cepa resistente de *E. coli* (USb007), con un promedio de tamaño de halo de 19 mm (cuadro 1).

Sin embargo, a diferencia de las pruebas del ensayo dual *in vitro,* no en todas las pruebas de actividad antimicrobiana con extractos etanólicos crudos se presentaron halos de actividad (cuadros 2 y 3). Las diferencias halladas con las pruebas de preselección dual podrían estar relacionadas con el hecho de que no todos los metabolitos con actividad antimicrobiana presentes en los hongos endófitos se extraen fácilmente con etanol, por lo cual es necesario extraerlos con otros disolventes orgánicos para recuperar todas las fracciones biológicamente activas. La baja actividad antimicrobiana también podría relacionarse con las concentraciones de los compuestos activos debidas a la cantidad de biomasa. Los extractos crudos filtrados podrían producir compuestos más potentes una vez se sometan a alguna purificación ^37^.

La identificación hasta el nivel de morfoespecie de los hongos endófitos fúngicos, permitió aislar e identificar en *M. americana* a *Bipolaris* spp., *Aspergillus* spp., *Penicillium* spp., *Cladosporium* spp. y *Mycelia sterilia,* las cuales se reportan por primera vez para esta especie vegetal en este trabajo. Algunos de los hongos endófitos aislados en *M. oleifera* han sido reportados previamente en otros estudios ^29^^,^^38^. Los hongos endófitos, como *Aspergillus* spp., *Cladosporium* spp. y *Mycelia sterilia,* comúnmente son aislados tanto en *M. oleifera* como en *M. americana* (cuadro 4). Los extractos fúngicos de los endófitos HEHMa6 *(Penicillium* spp.), HESMo8 *(Cladosporium* sp., 001) y HESMa10 *(Cladosporium* sp., 002), presentaron mayor actividad antibacteriana y, de forma coincidente, los dos aislamientos de los géneros *Cladosporium* se hicieron a partir de las semillas en las dos plantas utilizadas en este estudio.

En otras especies de plantas, se han aislado especies de *Cladosporium* spp.como hongos endófitos y se han reportado como fuente importante de antimicrobianos. Los metabolitos de *Cladosporium oxiosporum,* aislado de *Aglaia odorata* en Indonesia, exhibieron actividad antimicrobiana contra *E. coli, S. aureus* y *Candida albicans*^39^. En otro estudio, *Cladosporium uredinicola,* aislado de la fruta *Psidium guajava,* presentó actividad antimicrobiana contra *E. coli, S. aureus, Pseudomonas aeruginosa* y *Bacillus subtillis*^40^. Estos hallazgos demuestran la importancia de *Cladosporium* spp.como un hongo endófito de amplio espectro antimicrobiano.

Se ha aislado *Penicillium spp.* como endófito en otras especies de plantas, y se ha reportado su actividad antimicrobiana contra *E. coliy S. aureus*^41^, con halos de inhibición de 12 mm, siendo estos tamaños más pequeños que los hallados en el presente estudio. *Penicillium* spp. también se ha reportado como un hongo endófito de *M. oleifera*^42^, aunque ni su actividad antimicrobiana en cepas sensibles y resistentes de *E. coliy S.* aureu.s ni su aislamiento en la planta *M. americana,* se habían reportado antes.

En Colombia, la identificación y el aislamiento de hongos endófitos han sido escasos. Sin embargo, este tipo de estudio es de mucha relevancia debido a la gran biodiversidad de plantas medicinales del país, las cuales albergan un sinnúmero de comunidades microbianas endófitas con un gran potencial como fuentes naturales de antimicrobianos para el eventual desarrollo de fármacos.

Los resultados obtenidos en este estudio demostraron la diversidad de los géneros fúngicos de endófitos en *M. americana y M. oleifera, y* su potencial para la producción de nuevos antibióticos. La complejidad de la identificación de los hongos endófitos, las posibles variaciones morfológicas según los medios de aislamiento micológico y el gran número no cultivable, exigen la utilización de técnicas moleculares para seguir explorando la diversidad de los endófitos en estas plantas medicinales y continuar con el estudio de sus metabolitos como fuentes nuevas de antimicrobianos.
